# Nuclear mRNA Quality Control and Cytoplasmic NMD Are Linked by the Guard Proteins Gbp2 and Hrb1

**DOI:** 10.3390/ijms222011275

**Published:** 2021-10-19

**Authors:** Yen-Yun Lu, Heike Krebber

**Affiliations:** Abteilung für Molekulare Genetik, Institut für Mikrobiologie und Genetik, Göttinger Zentrum für Molekulare Biowissenschaften (GZMB), Georg-August Universität Göttingen, 37077 Göttingen, Germany; yen-yun.lu@biologie.uni-goettingen.de

**Keywords:** nuclear mRNA quality control, nonsense-mediated decay, RNA surveillance, splicing, guard proteins, Gbp2, Hrb1, SR proteins

## Abstract

Pre-mRNA splicing is critical for cells, as defects in this process can lead to altered open reading frames and defective proteins, potentially causing neurodegenerative diseases and cancer. Introns are removed in the nucleus and splicing is documented by the addition of exon-junction-complexes (EJCs) at exon-exon boundaries. This “memory” of splicing events is important for the ribosome, which translates the RNAs in the cytoplasm. In case a stop codon was detected before an EJC, translation is blocked and the RNA is eliminated by the nonsense-mediated decay (NMD). In the model organism *Saccharomyces cerevisiae*, two guard proteins, Gbp2 and Hrb1, have been identified as nuclear quality control factors for splicing. In their absence, intron-containing mRNAs leak into the cytoplasm. Their presence retains transcripts until the process is completed and they release the mRNAs by recruitment of the export factor Mex67. On transcripts that experience splicing problems, these guard proteins recruit the nuclear RNA degradation machinery. Interestingly, they continue their quality control function on exported transcripts. They support NMD by inhibiting translation and recruiting the cytoplasmic degradation factors. In this way, they link the nuclear and cytoplasmic quality control systems. These discoveries are also intriguing for humans, as homologues of these guard proteins are present also in multicellular organisms. Here, we provide an overview of the quality control mechanisms of pre-mRNA splicing, and present Gbp2 and Hrb1, as well as their human counterparts, as important players in these pathways.

## 1. Introduction

Correct gene expression is important for cells, and for the survival of multicellular organisms. Proper mRNA splicing ensures that mature transcripts carry the correct open reading frame (ORF) so that these sequences can then be translated into functional proteins. In addition, various regulated splicing events are used by organisms to fine-tune gene expression for specific needs (reviewed in [1,2]). Errors in splicing can cause deleterious life-threatening effects through the production of aberrant or unwanted proteins, or the depletion of required proteins. Splicing is thus quality controlled by systems that act both in the nucleus and in the cytoplasm to minimize aberrations in gene expression. In eukaryotes, splicing defects can lead to retention of transcripts in the nucleus instead of nuclear export, which eventually results in their degradation. In case the nuclear quality control fails, the transcripts that enter the cytoplasm can be caught by a second quality control system, the nonsense-mediated mRNA decay (NMD). This pathway captures mRNAs for which, for example, the splicing events resulted in premature termination codons (PTCs). These surveillance pathways must rely on specific quality control factors in addition to general RNA decay machineries to efficiently remove errors, but the details are not completely understood. Research using higher eukaryotic systems can be especially difficult due to the higher complexity of splicing events, which are furthermore influenced by the interplay and regulation of a great number of splicing factors and additional *cis*- or *trans*- regulatory elements (reviewed in [3]). The yeast *Saccharomyces cerevisiae* (hereafter referred to as yeast), with its relatively simple intronome but much conserved mechanisms for splicing, could therefore serve as a useful model organism for relevant research. Recently, studies in yeast revealed the involvement of two RNA-binding proteins, Gbp2 and Hrb1, in both the nuclear and cytoplasmic quality control of transcripts derived from intron-containing genes and thus show a link between both quality control pathways. These proteins share domain structural and functional similarities with the metazoan serine- and arginine-rich (SR) proteins, which are known to play important roles in splicing (reviewed in [4,5]). In this review, we briefly summarize current knowledge in eukaryotic mRNA quality control of spliced transcripts and highlight the newly proposed roles for Gbp2 and Hrb1. We discuss how these findings may offer insights for future exploration of yet unknown functions of mammalian SR proteins in the quality control of mRNA splicing and beyond.

## 2. Quality Control of Splicing in the Nucleus—Retention or Export of mRNA

Cells reduce harm from mRNA processing errors by preventing potentially problematic transcripts from entering the cytoplasm. Defects in splicing can lead to retention of transcripts at the transcription site, in specific nuclear domains, or at the nuclear pore complex (NPC). This correlates with the fact that splicing, as well as other processing steps, occur to a large extent co-transcriptionally and are intimately connected with mRNA export (reviewed in [6]). Delayed export allows more time for inefficient processing to complete, or is followed by the destruction of the transcripts by nuclear ribonucleases when further processing fails to succeed.

Inhibition of splicing has been found to cause retention of transcripts associated with stalled RNA polymerase II at chromatin [7,8]. Part of the effect is believed to be linked to improper mRNA 3′ end formation as splicing, especially of the last intron, affects the following cleavage and polyadenylation processes (reviewed in [9]), and impaired 3′ end processing also resulted in transcript retention at the transcription site [10,11,12]. Accumulating transcripts in the nucleus are mainly degraded by the nuclear exosome through its 3′–5′ exonucleolytic activity with the help of its cofactor, the TRAMP (Trf4/5–Air1/2–Mtr4 polyadenylation) complex (reviewed in [13,14]). In mammalian cells, the 5′–3′ exonuclease XRN2 was also found to play a role [15], although the detailed mechanism is less clear.

Studies in the mammalian system have demonstrated that improperly spliced transcripts that are polyadenylated are released from chromatin and accumulate in subnuclear domains termed nuclear speckles [8,16,17,18]. The mechanism by which transcripts are retained in these distinct domains is still unclear, but so far there is evidence indicating that phosphorylation of the arginine- and serine-rich (RS) domains of certain splicing factors and SR proteins as well as the regulated binding of mRNA nuclear export factor 1 (NXF1) and its adaptor proteins may be directly involved (reviewed in [19,20]). Interestingly, the accumulation of transcripts in nuclear speckles suggests that these mRNAs are not immediately targeted to degradation by the exosome, potentially due to protection from proper polyadenylation [20]. In fact, nuclear speckles were shown to be sites for post-transcriptional splicing [17,21,22] and it was proposed that transcripts are released and efficiently exported only upon complete maturation. Accordingly, several reports have demonstrated that mRNA nuclear retention due to retained introns can also be used as a mechanism for the temporal control of gene expression. Certain introns were found to be post-transcriptionally removed at distinct times, allowing regulated mRNA export and translation, which is especially relevant during cell differentiation [23,24,25].

RNA-binding adaptor proteins of the export factor TAP protein, also termed NXF1–NXT1 (or Mex67–Mtr2 in yeast), connect mRNA processing with nuclear export and are important players in nuclear retention of defective transcripts. They are recruited to the mRNA during processing and bind export factors depending on proper execution of the processing steps. Binding of export factors along the transcript is crucial for export as they coat the highly-charged mRNA and interact with the hydrophobic meshwork of phenylalanine–glycine (FG) nucleoporins in the interior of the NPC, allowing efficient passage of the messenger ribonucleoprotein (mRNP) [26,27]. In higher eukaryotes, splicing is often marked by the binding of an exon junction complex (EJC) upstream of the exon–exon junction [28]. Several SR proteins associate with the EJC and act as export adaptor proteins to facilitate nuclear export [29]. Research has shown that splicing alters the phosphorylation state of SR proteins, which in turn influences their binding to NXF1 [30,31,32]. It appears that SR proteins prevent the recruitment of NXF1 in their highly phosphorylated states to retain improperly spliced transcripts in the nucleus [20]. In yeast, although EJCs have not been identified, the SR-like proteins Gbp2 and Hrb1 similarly associate with the mRNA during splicing and bind Mex67 to support export [33]. Notably, upon splicing errors, Gbp2 and Hrb1 recruit instead the TRAMP–exosome complex, demonstrating a direct link between inhibition of export and degradation of the transcript. Another RNA-binding and export adaptor protein, Nab2, known to be involved in 3′ end processing, was also implicated in the quality control of splicing [34,35]. Overall, export adaptor proteins are important components of the nuclear mRNP and serve as checkpoints for the fidelity of pre-mRNA processing.

Quality control is additionally carried out at the NPC before the mRNP is transported into the cytoplasm. Several NPC-associated proteins were found to play a role, and one of the best-characterized factors among them is Mlp1, a yeast protein localized at the nuclear face of the NPC [27]. Mlp1 was shown to retain unspliced intron-containing transcripts in the nucleus through a mechanism that depends on the 5′ splice site [36]. The mammalian homolog, TPR, was also reported to regulate export of unspliced transcripts [37,38]. In addition, Mlp1 physically interacts with the export factor Mex67 and its adaptor proteins, which is thought to monitor completion of mRNA maturation, retaining those transcripts that are not properly packaged, hence likely not correctly processed [33,36,39,40]. Aberrant transcripts that are retained are degraded by the TRAMP–exosome machinery. In yeast, an endonuclease, Swt1, associates with the NPC and likely also plays a role in degrading defective transcripts [41,42].

Together, quality control of splicing in the nucleus occurs in parallel to the highly coupled transcription, splicing, and mRNA export pathways, and relies on key RNA-binding proteins that are central to the network of events.

## 3. Further Surveillance in the Cytoplasm—Detection of Errors via Nonsense-Mediated Decay

Despite surveillance in the nucleus, transcripts that carry defects resulting from splicing are found in the cytoplasm and can engage in translation. On one hand, quality control pathways are not perfect and faulty transcripts are provided with a chance to escape through the NPC [13,33,36,43,44], while on the other hand, incorrect splicing or intron retention via alternative splicing can lead to aberrations within the protein coding sequence, which are not recognized by nuclear quality control. However, these aberrations often generate premature termination codons (PTCs), and termination at a PTC activates the nonsense-mediated mRNA decay (NMD) pathway, which represses further translation of the transcript and trigger its degradation (reviewed in [45,46]).

Reports have shown that NMD is responsible for removing transcripts that are suboptimally spliced both in yeast and in higher eukaryotes [43,47,48,49]. Further, alternative splicing was estimated to commonly result in PTCs [50,51], and its coupling to NMD has been observed in several different species [52,53,54]. The link between alternative splicing and NMD is generally regarded as a mechanism to regulate the abundance of affected mRNAs in addition to surveillance, and has also been found to target several genes that encode factors involved in the splicing process per se (reviewed in [55]).

NMD is a well-conserved cytoplasmic mRNA quality control mechanism that has been found in species across the eukaryotic kingdom [45,46]. Although it was originally discovered to target PTC-containing transcripts, later research revealed that a large portion of normal, error-free mRNAs and some non-coding RNA species are also substrates of this pathway (reviewed in [56]). Thus, NMD functions beyond quality control and is now considered as a more general mechanism for post-transcriptional gene regulation [57,58].

The complete NMD pathway is carried out by factors specific to NMD as well as components of the general cytoplasmic mRNA decay machineries. Three specific proteins–Upf1, Upf2, and Upf3–were found to be conserved in all species tested and considered as core factors of NMD [46,59]. Among them, the ATPase-dependent RNA helicase Upf1 is the main factor that, with the support of Upf2 and Upf3, modulates target recognition and degradation. When the ribosome terminates at a PTC, Upf1 binds to the termination complex through interactions with the release factors eRF1 and eRF3 [60,61]. Upf2 and Upf3 join to form the Upf1–Upf2–Upf3 complex, which stimulates the enzyme activity of Upf1 [62]. Activated Upf1 translocates in the 5′ to 3′ direction on the target RNA to unwind secondary structures and dissociate bound proteins, including the ribosome, allowing complete degradation of the transcript [63,64,65,66,67]. In addition, Upf1 forms interactions with several other NMD and degradation factors, serving as a platform for the recruitment of these proteins [45,46].

The intimate relationship between splicing and NMD is illustrated by the involvement of EJCs in NMD target recognition. The presence of an EJC in the 3′ untranslated region (UTR) of a transcript downstream of a stop codon is the most prominent and efficient feature for NMD activation in higher eukaryotes [68,69]. The EJC consists of core factors eIF4A3, RBM8A, MAGOH, and is joined by multiple auxiliary factors, including SR proteins and UPF3 (reviewed in [70,71]). EJCs remain bound to the RNA throughout export and are normally dissociated by the translocating ribosome during the first round of translation [72]. However, since the ribosome disassembles at the stop codon and does not translate further into the 3′ UTR, any EJC that is in the 3′ UTR would generally not be removed and remain associated with the mRNA. Given that stop codons are typically present in the last exon, an EJC downstream of the stop codon implies that the stop codon is likely premature. In these cases, UPF2 is thought to be recruited to the EJC-bound UPF3 and subsequently interacts with UPF1 that associates with the terminating ribosome [62,73,74]. This would then activate UPF1, triggering downstream NMD effects. Therefore, EJCs can directly promote activation of NMD, exemplifying how nuclear splicing events communicate with cytoplasmic pathways through RNA-binding proteins for quality control. Interestingly, a recent study in yeast suggested that, despite lacking proteins homologous to EJC core components, an intron in proximity to a PTC enhances NMD [75]. This supports the possibility that a connection between splicing and NMD might be conserved from simple eukaryotic species.

Degradation of NMD targets in yeast depends mostly on the major 5′–3′ cytoplasmic exonuclease Xrn1 subsequent to decapping by Dcp1–Dcp2 and other cofactors [76,77,78,79,80]. Alternatively, substrates can be degraded from the 3′ end, albeit to a lesser extent, by the cytoplasmic exosome, supported by its cofactor, the Ski complex [79,81]. In higher eukaryotes, degradation of NMD targets additionally involves phosphorylation of UPF1 and several proteins encoded by the *smg* genes, originally identified in *Caenorhabditis elegans*. Thus UPF1, phosphorylated by the serine/threonine kinase SMG1 and its regulators, SMG8 and SMG9 [82], directly recruits SMG6, an endonuclease that cleaves the RNA at a site close to the termination codon [83,84,85]. The resulting 5′ and 3′ RNA fragments are subjected to degradation by the exosome and XRN1, respectively. Phosphorylated UPF1 also recruits the SMG5–SMG7 heterodimer [86,87], which subsequently recruits the CCR4–NOT complex for deadenylation [88]. Phosphorylated UPF1, SMG5, as well as the CCR4–NOT complex furthermore recruit the decapping complex, and the deadenylated and decapped transcript can then be degraded by XRN1 and the exosome.

Activation of NMD does not only lead to decay of the affected transcript, but also to a decrease in the translation efficiency of the targeted mRNA. It was shown in yeast that the Upf1-mediated identification of a reporter transcript as nonsense-containing induces translation repression and subsequently decapping [89]. Similarly, UPF1 was shown to decrease translation initiation of a reporter mRNA in human cells [90]. It was observed that phosphorylated UPF1 interacts with eIF3, a translation initiation factor that promotes the formation of active 80S ribosomes, and presumably in this way directly prevents further translation initiation.

Detection and elimination of PTC-containing transcripts by NMD is thought to serve as a fail-safe system for the quality control of spliced transcripts [91,92]. It was found that quite a surprising proportion of intron-containing transcripts escape the nucleus and are targeted to NMD in the cytoplasm [47,92]. Nuclear quality control in higher eukaryotes was likewise found to be leaky and insufficient for the elimination of all error-prone transcripts [13]. This rationalizes the need for surveillance systems in both cellular compartments. As a complicated pathway that involves multiple types of protein factors, the detail mechanisms of NMD have yet to be fully unraveled. The ongoing discovery of the widespread impact of NMD on gene expression and various diseases connected with defective NMD raises particular interest for the thorough understanding of this pathway.

## 4. Gbp2 and Hrb1 in Nuclear Quality Control of Splicing—Decay or Export of mRNA

Gbp2 and Hrb1 are two of the yeast RNA-binding proteins that shuttle between the nucleus and the cytoplasm. Both proteins consist of three RNA-recognition motifs (RRMs) as well as an SR/RGG domain at the N-terminus, which is rich in serine–arginine or arginine–glycine–glycine motifs [93,94]. A third yeast protein, Npl3, is also highly homologous and contains both RRM and SR domains [93,95]. These domain features resemble that of human SR proteins, a family of proteins typified by the presence of one or two N-terminal RRM domains and a C-terminal SR/RS domain [96]. Human SR proteins were initially discovered as key factors for constitutive and alternative pre-mRNA splicing, but in following research found to play more diverse roles in RNA metabolism (reviewed in [97]). The same holds true for the yeast SR-like proteins Gbp2, Hrb1, and Npl3, which were found to also participate in several RNA metabolic pathways [98,99,100,101,102,103,104,105] (see below). Interestingly, while all yeast SR-like proteins shuttle from the nucleus into the cytoplasm [93], only few of the human SR proteins move between these compartments [106,107]. This suggests that the shuttling species of human SR proteins likely share functional homology with the yeast SR-like proteins in addition to their domain structural similarities.

Gbp2 and Hrb1 join the journey of mRNAs in the nucleus, where they are co-transcriptionally recruited through the transcription/export (TREX) complex [93,108]. The TREX complex, comprising the THO complex (Tho2, Hpr1, Mft1, Thp2, Tex1), the DEAD-box helicase Sub2, and export factor Yra1, is recruited to the phosphorylated C-terminal domain of RNA polymerase II and functions to connect transcription with mRNA export by supporting the formation of an export competent mRNP [109,110]. Co-transcriptional recruitment of Gbp2 and Hrb1 is believed to contribute to transcription-coupled export, supported by findings that Gbp2 and Hrb1 bind to the mRNA export factor Mex67 and require it for their export [33,93,94].

Subsequent research indicated a role for Gbp2 and Hrb1 in nuclear quality control of splicing in addition to supporting export. First, it was observed that the co-transcriptional recruitment of Gbp2 and Hrb1 is also closely linked to splicing. Several reports have demonstrated that recruitment and stable binding of the THO/TREX complex is connected to splicing, both in yeast and in humans [111,112,113,114,115]. It was then shown that Gbp2 and Hrb1 co-purify with the spliceosome, specifically with the late splicing factors Prp17 and Prp43, and their binding to mRNAs depends on functional splicing [33,116]. This was further supported by transcriptome-wide analyses: the two proteins, in particular Gbp2, associate preferentially with transcripts that derived from intron-containing genes [33], and a transcriptome-wide binding profile of Gbp2 showed that it binds mRNAs mostly at the 5′ proximal region [117], which corresponds to the position of most yeast introns [118,119]. In another analysis using the PAR-CLIP method, Gbp2 showed distributed binding on mRNAs throughout the ORF, while Hrb1 showed a higher tendency to bind toward the 5′ end [120].

Functional splicing is further linked to the export of Gbp2 and Hrb1 with the mRNA, as binding of these proteins to Mex67 and their export to the cytoplasm were disrupted when splicing factor genes were mutated [33]. Interestingly, subsequent research revealed protein–protein interactions between Gbp2, Hrb1 and several factors involved in the nuclear quality control pathway: the helicase Mtr4 of the TRAMP complex, the 3′–5′ exonuclease Rrp6 of the nuclear exosome, and Mlp1 and Mlp2, two quality control factors at the NPC [33,121]. Moreover, a combination of *gbp2*Δ or *hrb1*Δ with mutation of the *MTR4* gene or deletion of the *RRP6* or *MLP1* gene resulted in synthetic sickness or lethality of the mutant cells [122]. These findings led to the idea that Gbp2 and Hrb1 may function in nuclear quality control, preventing transcripts that have not gone through proper splicing from being exported, and potentially facilitating their degradation by the TRAMP–exosome machinery. This was supported by results of mRNA leakage assays showing that faulty, intron-containing transcripts, which accumulated in the nucleus when *MTR4* or *RRP6* was deleted, were increasingly transported, or “leaked”, into the cytoplasm when Gbp2 or Hrb1 was also absent [33]. Furthermore, Gbp2 and Hrb1 showed increased binding to faulty transcripts when *RRP6* was mutated or when *MLP1* was deleted. These results imply that Gbp2 and Hrb1 retain improperly spliced transcripts in the nucleus for surveillance. The aberrant transcripts are likely directly eliminated by the nuclear exosome, agreeing with data showing that the TRAMP complex is recruited co-transcriptionally to intron sequences to facilitate splicing and quality control [123].

**Figure 1:**
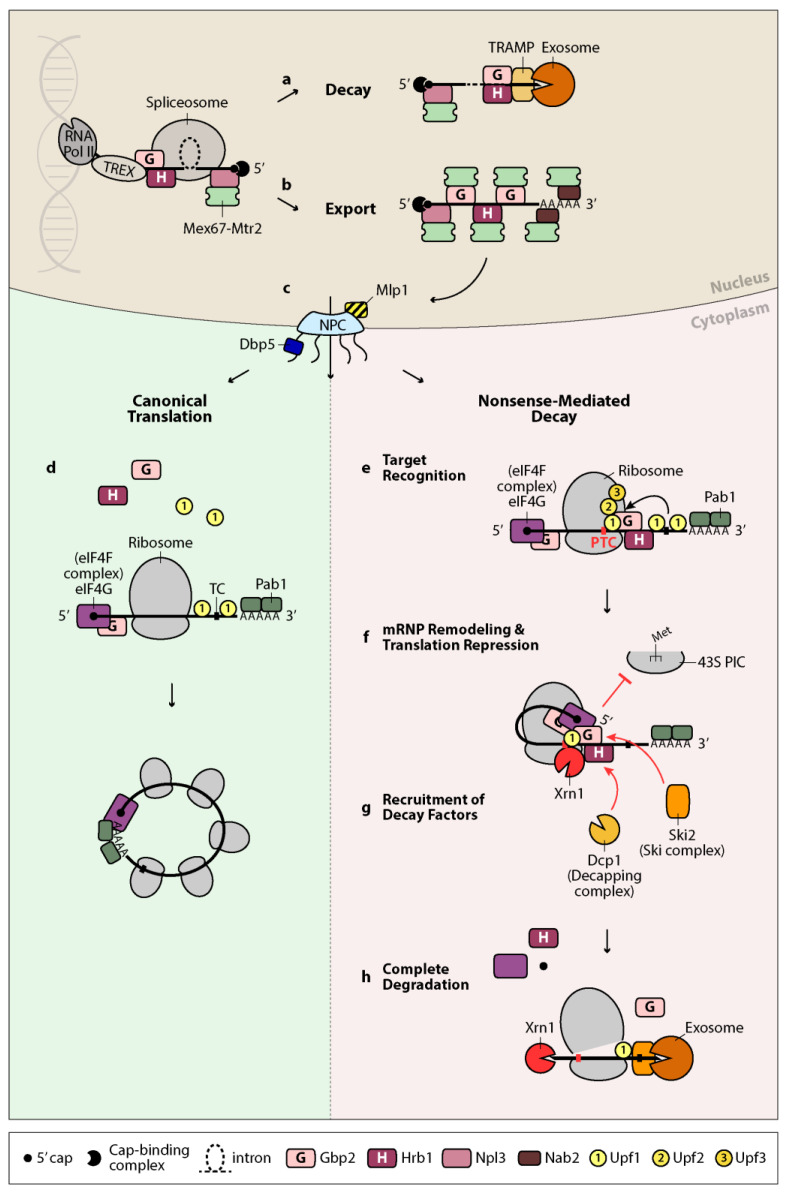
Model for the mRNA quality control functions of Gbp2 and Hrb1. Gbp2 and Hrb1 take part in mRNA quality control both in the nucleus and the cytoplasm. In the nucleus, they are loaded onto the nascent transcript through the TREX complex and associate with the late spliceosome. They recruit the TRAMP complex, which, upon errors in splicing, guides the faulty transcript to the nuclear exosome for degradation (**a**). In the event of correct splicing, Gbp2 and Hrb1 recruit instead the export factor heterodimer Mex67–Mtr2. Npl3 and Nab2 are two other RNA-binding proteins that associate with the mRNA co-transcriptionally. They also directly recruit Mex67–Mtr2, likely upon correct 5′ capping and 3′ polyadenylation, respectively, and together this supports proper packaging of the mature messenger ribonucleoprotein (mRNP) [27,127] (**b**). The mRNP is quality controlled by Mlp1 and other nuclear pore complex (NPC)-associated proteins before export (**c**). At the cytoplasmic side of the NPC, the helicase Dbp5 removes Mex67–Mtr2 and Nab2 from the emerging transcript, while Gbp2 and Hrb1 remain bound until translation. Correct transcripts undergo canonical translation, during which Gbp2 and Hrb1 are released (**d**). Efficient translation is thought to be supported by formation of a closed-loop RNA structure mediated by the interaction between 5′-associated eIF4G and poly(A)-binding protein Pab1 [128]. On PTC-containing transcripts, Upf1 associates with the terminating ribosome and is activated by the formation of the Upf1–Upf2–Upf3 complex (**e**). Gbp2 and Hrb1, potentially through dimer- or oligomerization, facilitate mRNP remodeling that brings the 5′ end of the transcript into proximity to Upf1, allowing direct contact of Upf1 with eIF4G. Gbp2 and Hrb1 bind to eIF4G and may repress translation initiation of the target transcript (**f**). Hrb1 promotes recruitment of decapping factor Dcp1, while both proteins promote recruitment of Ski2, a component of the cytoplasmic exosome cofactor Ski complex (**g**). The decapped RNA is degraded by Xrn1 from the 5′ end, while deadenylation and exosome-mediated decay occurs from the 3′ end. The helicase activity of Upf1 is required for ribosome disassembly and displacement of bound NMD factors, allowing complete elimination of the transcript (**h**). RNA Pol II: RNA polymerase II; TREX: transcription/export complex; NPC: nuclear pore complex; TC: termination codon; PTC: premature termination codon; 43S PIC: 43S pre-initiation complex.

A model was proposed for the nuclear quality control function of Gbp2 and Hrb1, suggesting that these proteins direct RNAs either towards decay or export (Figure 1a,b). They would interact with Mtr4 when the splicing process is somehow defective, leading to degradation of the transcript by the nuclear exosome. If splicing were carried out correctly, they would instead recruit Mex67–Mtr2 through interactions with Mex67, preventing degradation and promoting export. This was supported by studies showing that the presence of Gbp2 and Hrb1 was required for the association of Mtr4, and prevented the association of Mex67, with unspliced transcripts [33]. Surveillance occurs prior to export, as interactions between Gbp2, Hrb1, and Mex67 or Mlp1 were impaired when *MTR4* was mutated. Finally, the binding of Mtr4 and Mex67 to Gbp2 and Hrb1 was found to be mutually exclusive, supporting the “decay or export” notion. This is in line with a current model explaining how nuclear RNA exonucleases may selectively target aberrant transcripts. The model suggests that decay is the default pathway, which is kinetically competed against by proper processing, mRNP assembly, and timely export of the mRNA [13,124,125,126].

Notably, despite the involvement of Gbp2 and Hrb1 in the quality control of splicing, their absence did not lead to splicing defects and they are therefore not bona fide splicing factors [33,102]. Contrarily, the other yeast SR-like protein, Npl3, showed an impact on the splicing of numerous genes [102]. Subsequent research indicated that Npl3 interacts with and presumably promotes the recruitment of spliceosome assembly factors to support splicing, while Gbp2 and Hrb1 play a more specific role in detecting errors rather than affecting the process itself. The activities of Gbp2 and Hrb1 during co-transcriptional splicing and mRNA export provide mechanistic insights into the targeted degradation of aberrant transcripts in nuclear mRNA surveillance. Furthermore, this demonstrates that SR proteins associated during splicing may serve quality control functions for the process.

## 5. Gbp2 and Hrb1 in Nonsense-Mediated mRNA Decay—New Cytoplasmic Roles

Following nuclear quality control of splicing, Gbp2 and Hrb1 are transported to the cytoplasm along with the export of their bound mRNA. On the cytoplasmic side of the NPC, the DEAD-box helicase Dbp5 catalyzes the dissociation of Mex67, as well as Nab2, preventing the mRNP from diffusing back through the NPC and thereby providing directionality for mRNA export [127,129,130,131]. In contrast, Gbp2, Hrb1, and Npl3 remain bound and were found to co-purify with polysome fractions [105], suggesting that they have functions during translation.

Gbp2 and Hrb1 appear to participate in the NMD pathway, as their absence led to the accumulation of PTC-containing reporter transcripts both on the RNA and protein level in an Upf1-dependent manner [132]. Interestingly, the effect was only observed for reporters that were derived from intron-containing genes, but not for the traditionally used *PGK1*-based reporter, which does not contain an intron. This agrees with transcriptome-wide data showing that Gbp2 and Hrb1 are enriched on spliced transcripts [33]. Interaction and localization studies further indicated that Gbp2 and Hrb1 are physically associated with the NMD mRNPs on reporter and endogenous substrates that are undergoing degradation [132].

Based on the finding that the stable binding of Upf1 to an NMD reporter transcript was independent of Gbp2 and Hrb1, it was suggested that the two proteins do not play significant roles in NMD target recognition [132]. In contrast, the binding of Gbp2 and Hrb1 to the reporter decreased in *upf1*∆ cells compared to the wild type, indicating that they may be important for downstream processes of the pathway, and are removed as soon as the transcripts undergo translation. In fact, similar to their function in the nucleus, these proteins seem to facilitate degradation of the targeted transcripts by promoting the recruitment of decay factors. Hrb1 was shown to help recruit the decapping factor Dcp1, which is required for efficient 5′–3′ degradation by Xrn1 [132]. In addition, both Gbp2 and Hrb1 promoted the proper recruitment of Ski2, the helicase component of the cytoplasmic exosome cofactor Ski complex, and could thereby support degradation from the 3′ end. Interestingly, Ski2 is the cytoplasmic structural and functional homolog of Mtr4 [133], which was found to interact with Gbp2 and Hrb1 for nuclear mRNA quality control (see above). Participation of the two proteins in NMD substrate degradation corresponds with the findings that disruption of the 5′ degradation pathway led to prolonged association of the proteins with the substrate transcripts in the cytoplasm in an Upf1-dependent manner [132].

Besides RNA degradation, Gbp2 and Hrb1 may also serve specific functions in translation repression in NMD. Recent studies demonstrated that Gbp2 and Hrb1 show features similar to a group of yeast RGG motif-containing proteins that act as translation repressors [104,134]. These proteins inhibit translation initiation through their RGG motif-dependent interaction with eIF4G, a scaffold protein of the cap-binding eIF4F complex important for eukaryotic translation initiation (reviewed in [128]). However, unlike Gbp2 and Hrb1, the previously identified RGG motif-containing translation repressors did not show an effect on the NMD reporter transcript, pointing to the possibility that Gbp2 and Hrb1 could comprise specific activities in inhibiting translation initiation of their targeted NMD substrates [132]. Future investigation is required to reveal more details about their functions in translation repression for general or for specific transcripts.

Interestingly, Gbp2 and Hrb1 were also found to promote the direct interaction between Upf1 and eIF4G on the reporter transcript, which was thought to contribute to an efficient communication from PTC recognition to the 5′ end of the transcript, where downstream translation repression and target degradation initiates [132]. Given that the proteins can form dimers and potentially oligomers with themselves and each other, it was proposed that multiple molecules of these proteins at different positions of the transcript might interact and consequently result in RNA folding that brings the PTC-associated Upf1 in proximity to the 5′ end of the transcript. This mRNP restructuring model provides a mechanistic explanation for the rapid response of 5′ end degradation of NMD targets, which, as opposed to canonical 5′–3′ decay, is not dependent on prior deadenylation [79,135]. It may also explain how Upf1 physically interacts with numerous factors that participate at different stages of the pathway, as a favorable mRNP conformation would allow the main and auxiliary factors of early and late steps of NMD to conveniently come into contact as part of one bigger mRNP complex.

Although open questions remain, Gbp2 and Hrb1 are proposed to take part in the NMD pathway, likely on transcripts that have gone through splicing (Figure 1e–h). Upon target recognition, they may trigger mRNP remodeling and interact with eIF4G to repress translation initiation. Translation repression favors decapping, which is necessary for subsequent Xrn1-mediated degradation and supported by Hrb1-assisted recruitment of Dcp1. Gbp2 and Hrb1 may also help recruit the Ski complex and presumably, in turn, the cytoplasmic exosome for 3′ decay. In this way, Gbp2 and Hrb1 appear to continue their nuclear surveillance function in the cytoplasm and safeguard intron-containing transcripts in a more comprehensive manner, offering an extra level of protection to the cell.

## 6. Gbp2 and Hrb1 as Prototypes of Human Proteins

The accumulated effort in the functional characterization of Gbp2 and Hrb1 revealed multiple similarities between these proteins and the human SR proteins. In the nucleus, they are recruited to the transcript during transcription and are involved in splicing. Human SR proteins were found to actively regulate the splicing process, while Gbp2 and Hrb1 were found to act as surveillance factors. Through splicing, the proteins become preferentially associated with transcripts derived from intron-containing genes. Furthermore, Gbp2, Hrb1, and several SR proteins act as adaptor proteins for the export factors to facilitate mRNA export. In the cytoplasm, these proteins participate in NMD and are also implicated in regulating translation. Gbp2 and Hrb1 recruit decay machineries to support the efficient removal of NMD targets; SR proteins are auxiliary factors of the EJC and can promote NMD through EJC-dependent activation. Moreover, it was demonstrated that overexpression of the SR proteins SRSF1 and SRSF2 enhanced NMD, although this was not dependent on their shuttling activities [136]. Recently, it was shown that SRSF1 stimulates NMD by promoting Upf1 binding, and that SRSF2 affects the association of key NMD factors [137,138], demonstrating more direct links between SR proteins and cytoplasmic surveillance. Apart from NMD, Gbp2 can act as a translation repressor [134], whereas some SR proteins were shown to either suppress or enhance the translation of specific mRNAs [139,140]. An additional correlation is provided by the discovery of homologous proteins that phosphorylate these proteins—the yeast kinase Sky1 and its mammalian counterpart SRPK1 [141]—and mediate their import—the import receptors Mtr10 (in yeast) and TRN-SR (in Mammalia), respectively [142].

In light of these observations and the potential that SR and SR-like proteins still possess unidentified functions, we propose the idea that Gbp2 and Hrb1 may be the prototype of the EJC. Although present results do not show strict functional equivalences between these factors, Gbp2 and Hrb1 appear to serve as molecular marks for nuclear splicing and create a memory that can be linked to NMD in the cytoplasm, analogous to EJCs. This correlation of nuclear and cytoplasmic events enables communication between upstream and downstream processes and, importantly, provides the basis for various levels of regulation and surveillance. It is conceivable that a less distinguished form of the EJC suffices for the relatively simple genome and modest intronome in yeast. As the complexity of gene expression increased through evolution, the functions of Gbp2 and Hrb1 may have elaborated into the larger family of SR proteins and their more specialized and specific activities in splicing, accommodating, and also creating more possibilities for regulation. In addition, the complex nature of alternative splicing results in high rates of errors [49,143], which may have prompted a stronger link between splicing and NMD to improve the stringency of surveillance, implemented through the direct role of EJC in NMD activation. Accordingly, the quality control functions of Gbp2 and Hrb1 are most likely conserved and may hint to the homologous activities of EJC-associated human proteins. Future studies are expected to reveal more similarities as well as differences between the yeast SR-like proteins and their human counterparts, which would offer a more complete picture of how the significance and versatility of these RNA-binding proteins have developed early but continued to advance through evolution, serving crucial roles in the regulation of gene expression.

## 7. Closing Remarks

Splicing is an essential mechanism that shapes the diverse but carefully controlled output of the genome. It comes as no surprise that misregulation of splicing events underlies a myriad of severe abnormalities found in organisms, including developmental disorders, neurodegenerative and inherited diseases, as well as cancers (reviewed in [144,145,146]). Recently, alternative splicing has also been implicated in aging and longevity (reviewed in [147]). Many studies have therefore strived to develop therapeutic strategies that focus on splicing modulators and the regulators of these factors. Continued research on the molecular mechanism of splicing and its quality control pathways further deepens our knowledge and may support the identification of novel therapeutic targets.

The family of SR proteins plays critical roles in splicing, but the range of their cellular activities is still expanding. For example, Gbp2 and several human SR proteins were found to accumulate in stress granules or distinct cytoplasmic foci under specific types of cellular stress, and were suggested to participate in translation repression under these circumstances [134,148,149]. In addition, transcriptome-wide analysis of yeast RNPs has revealed that Gbp2 is also strongly associated with small nucleolar RNAs and long non-coding (lnc) RNAs [117]. Subsequent reports demonstrated that lncRNAs engaged in translation could be targeted to NMD, and this is functionally significant for the regulation of gene expression [56,68,150,151]. Recent reports have further implicated a regulatory interplay between human SR proteins, microRNAs, and lncRNAs underlying oncogenic or other health effects [152,153,154,155,156]. Unraveling of the complete repertoire of events in which SR proteins participate is anticipated, and will hopefully bring about exciting and fruitful development in disease curing.
